# Lymphedema following cancer therapy in Slovenia: a frequently overlooked condition?

**DOI:** 10.2478/v10019-010-0047-3

**Published:** 2010-11-16

**Authors:** Tanja Planinsek Rucigaj, Nada Kecelj Leskovec, Vesna Tlaker Zunter

**Affiliations:** Department of Dermatovenereology, University Medical Center Ljubljana, Ljubljana, Slovenia

**Keywords:** lymphedema, secondary lymphedema, cancer therapy, adverse effects, radiotherapy, adverse effects

## Abstract

**Introduction:**

Secondary lymphedema following cancer therapy is a frequent, often painful, quality of life disturbing condition, reducing the patients’ mobility and predisposing them to complications, e.g. infections and malignancies. The critical aspect of lymphedema therapy is to start as soon as possible to prevent the irreversible tissue damage.

**Patients and methods:**

We performed a retrospective study of patients with lymphedema, treated at the Department of Dermatovenereology, University Medical Center Ljubljana, from January 2002 to June 2010. The patients’ demographic and medical data were collected, including type of cancer, type and stage of lymphedema, and time to first therapy of lymphedema. The number of referred patients with lymphedema following the therapy of melanoma, breast cancer, and uterine/cervical cancer, was compared to the number of patients expected to experience lymphedema following cancer therapy, calculated from the incidence reported in the literature.

**Results:**

In the period of 8.5 years, 543 patients (432 females, 112 males) with lymphedema were treated. The results show that probably many Slovenian patients with secondary lymphedema following cancer therapy remain unrecognized and untreated or undertreated. In the majority of our patients, the management of lymphedema was delayed; on average, the patients first received therapy for lymphedema 3.6 years after the first signs of lymphedema.

**Conclusions:**

To avoid a delay in diagnosis and therapy, and the complications of lymphedema following cancer therapy, the physician should actively look for signs or symptoms of lymphedema during the follow-up period, and promptly manage or refer the patients developing problems.

## Introduction

Lymphedema is swelling due to excess accumulation of lymph in the tissues caused by inadequate lymph drainage. Lymphedema differs clinically form other forms of chronic edema by its altered skin texture and brawny quality of the subcutaneous tissues which limit pitting. Primary lymphedema implies a genetic or constitutive cause whereby there is an intrinsic fault in the lymph drainage determined by the lymphatic maldevelopment or functional weakness. Secondary lymphedema implies an acquired failure of previously normal lymph drainage due to an identifiable cause extrinsic to the lymphatic system.^1^ Secondary types of lymphedema are more common than the primary ones. In developed countries, cancer treatment dominates as a cause, whereas in tropical climates lymphedema is usually due to infections.^2^

The reports on worldwide incidence of lymphedema following cancer therapy in the available literature are scarce and varying considerably. The estimated incidence of breast cancer-related lymphedema is ranging from 13–50%.^3^–^5^ The introduction of sentinel lymph node biopsy reduced the incidence of lymphedema, however not as dramatically as it was hoped.^1^ The incidence of lower limb lymphedema following radical hysterectomy alone is estimated at 5–10% but can be as high as 49% by 10 years of follow-up in patients who also received the adjuvant radiation treatment.^6^–^7^ The incidence of lymphedema after lymphadenectomy for melanoma can be up to 44% after therapeutic groin dissection for palpable disease,^8^ but the incidence following sentinel lymph node biopsy is much less.^7^,^9^

There are no available epidemiological reports of lymphedema following cancer surgery in Slovenia. A decade ago, an outpatient office specialized in lymphedema was introduced at the Department of Dermatovenerology, University Medical Center Ljubljana. As this is the only specialized center of this type in Slovenia, the number of referred patients is constantly increasing. Besides providing the best of care for these patients, one of the future aims of the center is to establish a national registry of lymphedema to provide epidemiologic data.

The clinical staging of lymphedema is shown in Table 1. Latent or subclinical (stage 0) lymphedema, even after surgical lymphadenectomy, may persist for months to years without any clinical evidence of lymphatic disturbance. Trigger events, e.g. insect sting, physical exertion, injuries, inflammation or warming of the limb may cause edema, which is either reversible or may, with additional lymphatic overload, proceed to the following stage. In stage I, the edema is reversible, soft, disappearing spontaneously overnight or, with compression therapy, during the day. The skin is smooth, with small pits. Stage I may persist for several years. However, if left untreated, it sooner or later proceeds to the chronical stage II.

During the stage II, edema persists despite limb elevation. In the early stage, edema is still pitting, later the edema is non-pitting, elastic. The skin feels harder, fibrotic. This phase cannot be reversed spontaneously without therapy. The Stemmer’s sign is positive – the skin on dorsal surface of the second toe cannot be pinched into a fold.

During the stage III (*elephantiasis*) the edema is enormous. The skin shows trophic changes (fibrosis, hyperkeratoses, papillomatosis, hyperpigmentations, lymphorea, ulcerations) and is prone to bacterial and fungal infections. The condition may only partly improve with the appropriate therapy. Occasionally, development of highly malignant lymphangiosarcoma or other malignancies may ensue.^1^,^10^–^12^

Pain may be present during all stages. The patients also report numbness, feeling of heaviness, fatigue, paresthesias, or mobility disturbances of the affected limb.^10^–^12^

Irreversibility of the later stages of lymphedema calls for timely therapeutic intervention. The delay in seeking medical attention for lymphedema by the patient, as well as the physicians’ unawareness or underestimation of the condition might lead to chronic, hard to manage problems. During follow-up after cancer surgery and/or radiotherapy, the physician should actively look for signs or symptoms of lymphedema, and promptly manage or refer the patient developing problems.

As in case of other side effects following cancer therapy, the therapy of lymphedema should be tailored individually.^13^ It should consider the patient’s clinical situation, history, and any coexisting illnesses. The patient’s compliance is of crucial importance. Therefore, the continuous patient education and encouragement are essential parts of the management.

Edema should be reduced as early as possible, using the compression therapy and/or manual lymph drainage. During improvement, compression stockings are required to maintain the improved condition.^11^,^12^,^14^–^17^

The opinions and studies on drug therapy for lymphedema are controversial. Invasive approaches may be appropriate only in a minority of patients. Surgery may cause further damage to lymphatics, and lead to ulceration, fistulas, skin necrosis and exacerbation of edema.^12^

The recommended additional measures include mobilization to improve the muscle pump function. Extreme heat, cold, and trauma should be avoided. Proper skin care to prevent infections is also an important part of the management.^11^

## Patients and methods

We performed a retrospective study of patients with lymphedema treated at the Department of Dermatovenereology, University Medical Center Ljubljana, from January 2002 to June 2010. The patients’ charts were reviewed, and the following data were collected: demographic data, type of malignancy, year and type of the oncologic procedure, type, location and stage of lymphedema, time of appearance of lymphedema after the procedure, and duration of lymphedema before the first intervention. The average time of appearance of lymphedema after the procedure and the average duration of lymphedema before the first intervention were calculated by using simple statistical methods. The expected incidence of lymphedema following melanoma, breast cancer, and uterine/cervical cancer was calculated from the incidence reported in the Slovenian cancer registry in the years 2002–2007^18^, and compared to the worldwide lymphedema incidence reports^4^–^9^, by using simple statistical methods.

## Results

In the period of 8.5 years, 543 patients (432 females, 112 males) with lymphedema were treated. Of the 543 patients, 139 patients presented with primary and 404 with secondary lymphedema in 776 localizations. Secondary lymphedema following cancer therapy was found in 227 patients. Details are shown in Tables 2 and 3.

The average time from cancer therapy (surgery or/and radiotherapy) to the development of lymphedema was 3.4 years. In 112 patients, edema started shortly after the procedure, however, maximal time to the development of lymphedema was 31 years after the cancer therapy in one patient. The average time from the appearance of lymphedema to start of the therapy for lymphedema was 3.6 years. Only three patients received therapy for lymphedema as soon as the edema started, and maximal time from beginning of lymphedema to therapy was 28 years. The average time from cancer intervention to start of lymphedema therapy was 7 years, maximum 39 years. Only one patient received the therapy for lymphedema immediately after the procedure for cancer.

The causes of lymphedema in our oncologic patients are shown in Table 4, by the year of the first referral.

The number of patients referred for lymphedema following cancer therapy for melanoma, breast cancer, and uterine/cervical cancer from 2002 to 2007, and the comparison to the expected incidence of lymphedema is shown in Table 5.

## Discussion

The reports on the worldwide incidence of lymphedema following cancer therapy in the available literature are scarce and varying considerably. Until present, there were no available epidemiological reports of lymphedema following cancer surgery/radiotherapy in Slovenia.

The majority of patients with secondary lymphedema following cancer referred to Department of Dermatovenerology during the observed period were patients with lymphedema of the lower extremities following uterine/cervical cancer, patients with lymphedema of the upper extremity following breast cancer, and patients following lymphedema after procedure for melanoma, accounting together for 77.5% of all patients with secondary lymphedema following cancer. The management of lymphedema in the included patient population was delayed; the patients first received therapy for lymphedema on average 3.6 years after the first signs of lymphedema and on average 7 years after the procedure for cancer.

Calculated from the reported incidence of cancer in Slovenia^18^ and the reported worldwide incidence of lymphedema due to cancer^4^–^9^, the expected incidence of lymphedema in Slovenia during the period 2002–2007 following melanoma, breast cancer, and uterine or cervical cancer is much higher than the actual number of patients referred to the Department of Dermatovenerology during this period. Since the Department features the only specialized center for lymphedema in Slovenia, it can be possibly concluded that many patients with lymphedema after cancer therapy remain unrecognized and untreated or undertreated.

Beside the adequate choice of the oncological treatment option^19^,^20^, the physician should actively look for signs or symptoms of lymphedema during the follow-up of patients. If lymphedema after cancer surgery and/or radiotherapy is observed, the patient developing problems should promptly be referred. Lymphedema during the early stages is a reversible or partially reversible state, whereas it is irreversible and very hard to manage if left to proceed to the late stages, causing great patient disability and predisposing them to complications, eg. infections and malignancies. The results of the present study also emphasize the need to establish a Slovenian national registry of lymphedema.

## Figures and Tables

**TABLE 1.** Staging of lymphedema

**Stage**	**Clinical findings**	**Stemmer’s sign**
0	Latent phase – no edema	Negative
I	Soft edema	Negative
II	Initially: pitting edema; Longstanding: elastic edema	Positive
III	Hard, enormous edema, skin changes	Positive

**TABLE 2.** Number of patients by type of edema

**Total: 543 patients (432 females, 112 males)**		
Primary lymphedema 139 patients	Secondary lymphedema 404 patients	
	Following cancer therapy 227 patients (198 females, 29 males)	Due to other causes 177 patients

**TABLE 3.** Lymphedema by localizations

**776 localizations of lymphedema in 543 patients**	
498 localizations of lymphedema due to a non-malignant cause	278 lymphedemas following a malignant disease
	199 edemas of the lower extremity
	72 edemas of the upper extremity
483 edemas of the lower extremity	2 facial edemas
15 edemas of the upper extremity	2 trunk edemas
	1 scrotal edema
	1 penile edema
	1 breast edema

**TABLE 4.** Malignancies that caused lymphedema in our patients, by the year of first referral

	**Year**									

Cause	2002	2003	2004	2005	2006	2007	2008	2009	2010 (Jan–Jun)	Total
Breast cancer	1	2	2	12	6	3	6	22	14	68
Uterine and cervical cancer	6	9	13	8	9	7	3	11	1	67
Melanoma	2	3	2	3	4	6	4	10	7	41
Sarcoma	1	1	1	1	1	1	1	2	1	10
Colorectal cancer	1	2	0	0	0	2	0	2	1	8
Post-radiotherapy for lymphoma	0	1	1	1	1	1	1	0	1	7
Vulvar cancer	0	0	1	2	0	1	1	1	0	6
Prostatic cancer	0	1	0	0	0	0	0	2	2	5
Ovarian cancer	1	0	0	1	0	0	2	0	0	4
Lymphadenectomy for unknown cancer	1	0	0	0	0	1	1	1	0	4
Testicular cancer	0	0	1	1	0	0	0	0	1	3
Bladder cancer	0	0	1	0	0	0	0	1	0	2
Lung cancer	0	0	0	0	1	0	0	0	0	1
Non-melanoma skin cancer	0	0	0	0	0	0	1	0	0	1
Total	13	19	22	29	22	22	20	52	28	227

**TABLE 5.** Slovenian total incidence of melanoma, breast cancer, and uterine/cervical cancer in 2002–2007^18^, compared to the number of patients expected/referred for lymphedema after procedure for cancer. *Expected number of patients with lymph-edema after therapy for the relevant condition, according to the published reports^3^–^9^

	**Year**					
	**2002**	**2003**	**2004**	**2005**	**2006**	**2007**
**Melanoma**						
Total incidence	148	202	210	226	395	437
Expected patients with lymphedema (N)*	65	88	92	99	173	192
Referred patients with lymphedema (N)	2	3	2	3	4	6
**Breast cancer**						
Total incidence	817	856	911	944	1117	1153
Expected patients with lymphedema (N)*	106–408	111–428	118–455	122–472	145–558	149–576
Referred patients with lymphedema (N)	1	2	2	12	6	3
**Uterine/cervical cancer**						
Total incidence	449	467	476	480	442	449
Expected patients with lymphedema (N)*	22–220	23–228	24–233	24–235	22–216	22–220
Referred patients with lymphedema (N)	6	9	13	8	8	7
